# Draft Genome Sequence of Labrys okinawensis, Isolated from *Acaciella* sp. Nodules in Mexico

**DOI:** 10.1128/mra.00732-22

**Published:** 2022-11-07

**Authors:** Belén Chávez-Ramírez, Violeta Larios-Serrato, Paulina Estrada-de los Santos

**Affiliations:** a Instituto Politécnico Nacional, Escuela Nacional de Ciencias Biológicas, Alcaldía Miguel Hidalgo, Mexico City, México; SIPBS, University of Strathclyde

## Abstract

A new plant-associated bacterium, Labrys okinawensis strain LIt4, was isolated from root nodules of wild *Acaciella* sp. in Morelos, Mexico. The 6,499,737-bp genome sequence provides opportunities to investigate a new reference strain to add information about the species *L. okinawensis*.

## ANNOUNCEMENT

The publication of new species is frequently performed with a description of a single strain. This was the case for Labrys okinawensis, first isolated from the root nodules of Entada phaseoloides in Okinawa, Japan (1). The description in this study of a new *L. okinawensis* strain provides more information about the divergence, ecological, and geographical distribution of the species. Strain LIt4 was isolated from the root nodules of *Acaciella* sp. in Morelos, Mexico (18°54′20.999″N, 99°1′28.999″W) (2). Briefly, nodules were randomly selected, washed, disinfected in ethanol for 30 s, immersed in 10% commercial chloride for 10 min, and rinsed 5 times with sterile water. The nodules were crushed and streaked onto yeast extract-mannitol medium containing 5 g mannitol. The plates were incubated at 28°C for 3 to 5 days. A colony was selected and streaked onto fresh plates to obtain an axenic culture.

**FIG 1:**
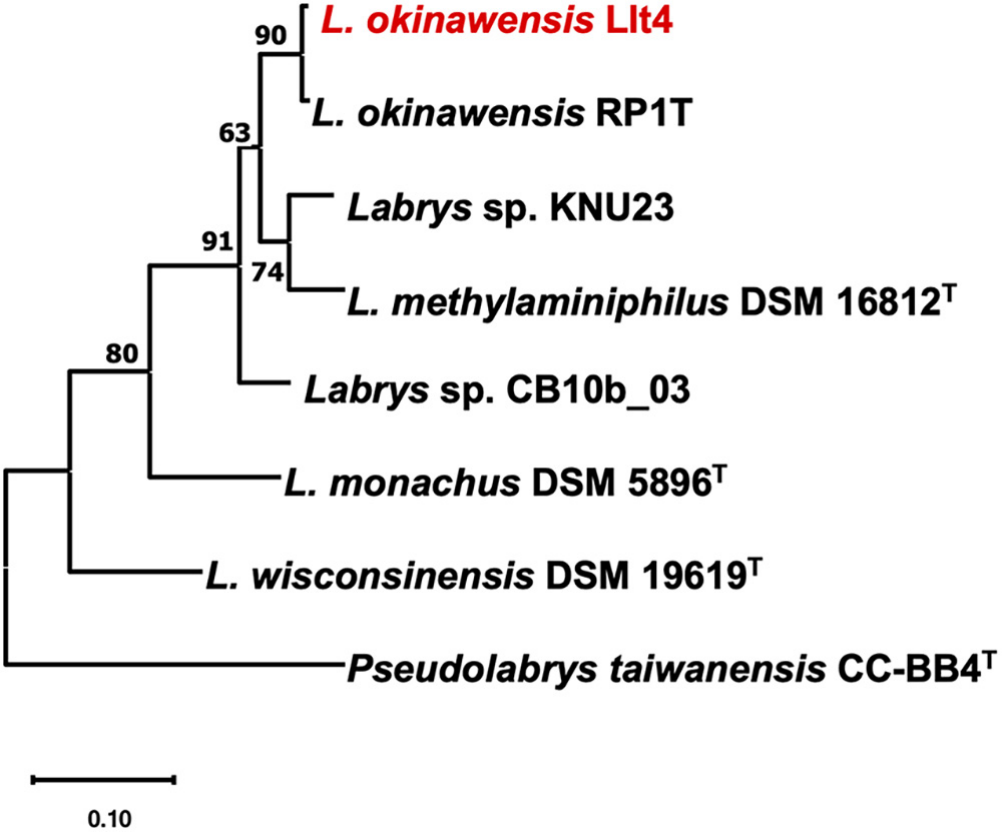
Phylogenomic analysis of *Labrys* species. The tree was inferred with up-to-date bacterial core genes (UBCG pipeline), with concatenated alignment of 92 core genes. The tree was determined using the maximum likelihood reconstruction method and the GTR+CAT model. Gene support indices (GSI) and percentage bootstrap values are given at the branching points. A total of 92,586 nucleotide positions were used. The bar shows the number of substitutions per position. The species in this study appears in red.

Genomic DNA was isolated using the cetyltrimethylammonium bromide (CTAB) method (3). The genome sequence was obtained by Novogene using the Illumina NovaSeq 6000 platform (150-bp paired-end reads; mean read length, 150 bp) with the NEBNext Ultra DNA library prep kit and an insert size of 350 bp. A total of 9,186,340 raw reads were obtained. Default parameters were used for all software unless otherwise described. A quality control analysis of the assembly was performed using FastQC v0.11.9 (https://www.bioinformatics.babraham.ac.uk/projects/fastqc/), checking for adapters and the length of the reads. Trimming and filtering of the information was performed using Trimmomatic v0.39 (4), keeping only those sequences with a Phred quality of >28 and filtering sequences of >36 bp. All sequences were assembled *de novo* using the SPAdes v3.14 program (5). The resulting scaffolds.fasta file underwent a cleaning process to remove spurious sequences using BLASTN v2.9.0 (6). For the cleaning step, a database was created using five *Labrys* genomes available in the NCBI Genome database (GenBank accession numbers GCA_002216255.2, GCA_008330945.1, GCA_017813775.1, GCA_001676615.1, and GCA_002982075.1). The scaffolds from strain LIt4 were aligned with those in the *Labrys* genome database (7); those scaffolds with E values of >0.00001 were eliminated. Spurious sequences (163) were removed, many of them shorter than 500 bp. Additionally, in mid-November 2020, VecScreen (https://www.ncbi.nlm.nih.gov/tools/vecscreen/) was accessed to detect and exclude one sequence with 28 bp. Metrics like the *N*_50_ value and misassemblies were obtained using QUAST v5.0.2 (8). Annotation was performed using the standard operating procedure at the NCBI Prokaryotic Genome Annotation Pipeline (PGAP) v5.1. The draft genome sequence contained 134 scaffolds (*N*_50_, 323,395 bp), with 6,499,737 bases and a GC content of 64.07%. A total of 5,978 genes were obtained, with 5,886 protein coding genes, 56 rRNAs, and 49 tRNAs. Using FastANI v0.1.3 (https://www.kbase.us/), the average nucleotide identity was calculated, resulting in 98.6% identity to *L. okinawensis* RP1T (NZ_PUEJ01000010.1). A phylogenomic analysis was computed using the up-to-date bacterial core genes (UBCG pipeline v2.0) (9), showing the insertion of strain LIt4 into the genus *Labrys* and grouping it with *L. okinawensis* (Fig. 1). Strain LIt4 was predicted to lack *nifH* and *nodC*, suggesting a nodule-associated bacteria (10).

The addition of new reference strains to a described species that contains only a few strains is essential to know more about the distribution of a species.

### Data availability.

The draft genome sequence of strain LIt4 has been deposited at NCBI GenBank under the accession number JAGIPY000000000. The BioProject and BioSample accession numbers are PRJNA717099 and SAMN18477566, respectively. The SRA accession number is SRX16249345.
